# Rheological Characterization of Yeast Protein–Sanxan Composite Hydrogels via SAOS, LAOS and Thermal Analysis

**DOI:** 10.3390/gels12080747

**Published:** 2026-08-20

**Authors:** Xuesong Cao, Yujie Qu, Zhiping Fan

**Affiliations:** 1 Medical School, Shandong Xiehe University, Jinan 250109, China; 2 Institute of BioPharmaceutical Research, Liaocheng University, Liaocheng 252059, China

**Keywords:** yeast protein, sanxan, hydrogel, rheology, LAOS

## Abstract

Future foods are driving an urgent need for sustainable and functional protein resources, and synthetic biology is emerging as a powerful platform to produce such proteins efficiently. Here, we designed a yeast protein (YP)–sanxan composite hydrogel obtained. The introduction of YP significantly improved thermal stability (by 5–15 °C) and ensured polymer compatibility. Rheological analysis indicated a frequency-dependent weak gel (tan δ = 0.1–0.3), making it suitable for safe swallowing. The material exhibited Type III nonlinear viscoelastic behavior, characterized by inter-cycle strain softening and a weak overshoot in G″, with Lissajous curves revealing a strain-induced transition from solid-like to fluid-like behavior. Crucially, YP-reinforced gels (5–20%) exhibited higher elastic moduli, indicating that the incorporation of YP strengthened the gel network and increased its structural rigidity, as further confirmed by Strain Sweep. With its tunable rheology and superior thermal stability, this hydrogel holds great potential for functional foods, 3D food printing, delivery systems, and biomedical scaffolds.

## 1. Introduction

As an interdisciplinary frontier science, the core objective of synthetic biology is to design, construct, or reprogram biological systems through an engineering mindset, thereby endowing them with novel functional characteristics [1]. This field primarily relies on key technologies such as gene editing, DNA synthesis and assembly, and gene circuit design, aiming to build “life machines” with high predictability and operability [2,3]. It has demonstrated broad application prospects across numerous sectors, including medicine and health (engineered cell therapies, novel drug production), sustainable energy and chemicals (biofuels, biomaterials), agriculture and food (crop improvement, alternative proteins), and environmental protection (pollutant degradation).

Driven by global population growth and the evolution of dietary structures, the demand for protein continues to increase [4]. Traditional protein sources primarily include animal-derived proteins (pork, beef, and chicken) and plant-derived proteins (soy and wheat). Although plant-based proteins are lower in cost and abundant in reserves, certain bioactive components they contain may trigger adverse reactions in humans. This presents an opportunity for the development of novel alternative proteins. Yeast protein (YP), as a microbial-derived protein, can be consumed directly or utilized as a raw material in food processing due to its reliability, stability, and sustainability [5,6]. Notably, essential amino acids account for up to 40% of YP, and its amino acid profile closely resembles that of whey protein—an ideal reference protein [7]. However, industrially produced YP exhibits a dense structure, making it difficult to form stable gels or systems when used alone [8]. This limitation restricts its application in gel-based products [8]. Research indicates that introducing polysaccharides can form composite gel systems with proteins through molecular interactions like hydrogen bonding [9,10]. Sanxan, an anionic heteropolysaccharide with unique physicochemical properties (high freeze–thaw stability, hydrophilicity, and tunable rheology) [11], was approved as a food additive by the China National Center for Food Safety Risk Assessment (CFSA) in 2020. This provides crucial material support for developing novel emulsion or gel products. Among commonly employed food polysaccharides such as xanthan gum (shear-thinning but non-gelling), gellan gum (thermoreversible but rigid), and carrageenan (thermally irreversible), Sanxan stands out as a novel polysaccharide with distinct thermoreversible gelation behavior. Unlike xanthan gum, which primarily imparts viscosity without network formation, Sanxan forms elastic, self-recovering gels upon cooling, enabling superior textural control in low-concentration food systems. Its repeating tetrasaccharide unit composed of D-mannose, D-glucuronic acid, L-rhamnose, and D-glucose exhibits structural homology to gellan gum, yet demonstrates enhanced thermal stability and recovery kinetics under dynamic processing conditions. These properties position Sanxan as a uniquely suitable matrix for developing next-generation functional hydrogels with tunable thermo-responsiveness, particularly in low-sugar or thermally processed food applications [12,13]. Incorporating Sanxan addresses the gelation limitation of YP by providing a physical crosslinking scaffold. The synergistic interactions between Sanxan and YP may form a continuous three-dimensional network that enhances structural stability, similar to networks reported in other gel systems [14,15,16].

In the context of sustainable development and the burgeoning market for functional food materials, this research focuses on engineering a novel Sanxan–Yeast Protein (SYPs) composite gel system exhibiting superior functional attributes. This study addresses the following question: How does YP concentration regulate the thermal stability and the transition from linear to nonlinear viscoelastic behavior in sanxan-based gels? We hypothesized that YP interacts with sanxan through non-covalent protein–polysaccharide associations, thereby reorganizing and reinforcing the gel network. Such concentration-dependent network modification was expected to increase the elastic modulus and critical strain while producing distinct changes in energy dissipation. To test this hypothesis, composite gels containing different YP and sanxan concentrations were systematically characterized using TGA, DSC, SAOS, amplitude sweeps, LAOS, Lissajous analysis, and S-T factor mapping.

## 2. Result and Discussion

### 2.1. TGA and DSC

**Figure 1:**
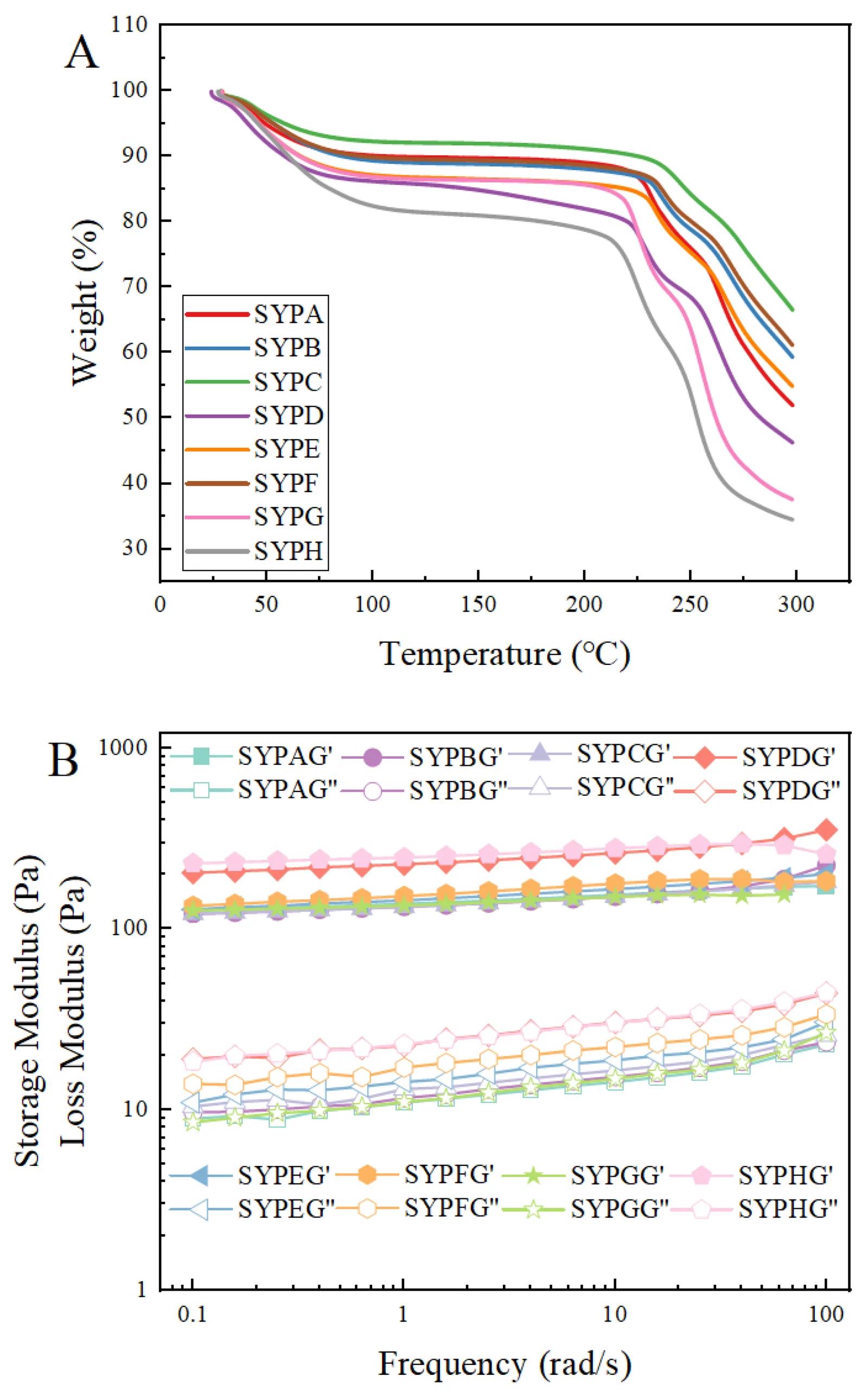
(**A**) TGA curves of SYPs hydrogel; (**B**) frequency sweep curves within the range of 0.1–100%.

To elucidate the application potential of the composite hydrogel under extreme thermal conditions, the thermal properties of SYPs samples were systematically evaluated using TGA and DSC (Table 1). Figure 1A presents the TGA profiles of the freeze-dried gel samples across a temperature range of 30–300 °C. The thermograms revealed an initial mass loss near 100 °C, which was principally ascribed to the evaporation of adsorbed free water. A second, more pronounced mass loss was observed in the 210–230 °C interval. This degradation event corresponded to the thermal decomposition of the gel matrix, signifying that elevated temperatures compromised the material’s structural integrity. Notably, the interaction between YP and Sanxan varied significantly with concentration, manifesting as distinct levels of thermal stability enhancement. TGA analysis of the pure YP (30–400 °C, Figure S1) corroborated the intrinsic ability of the YP component to bolster the thermal stability of the gel system. Comparative analysis revealed that YP incorporation substantially enhanced the thermal stability of composite gels relative to pure sanxan, elevating degradation temperatures by approximately 5–15 °C. Specifically, within the low-concentration sanxan group, composite gels demonstrated increases of approximately 5–13 °C compared to the blank control SYPG. Similarly, in the high-concentration sanxan group, degradation temperatures exceeded those of the corresponding blank control SYPH by approximately 6–15 °C.

The thermal behavior of the samples was evaluated using DSC over a temperature range of −30 °C to 40 °C. As evidenced by the data and Figure S2, the incorporation of YP did not significantly compromise the cryogenic performance of the gel matrix. The preservation of low-temperature stability upon protein addition markedly improved the environmental robustness and broadened the application prospects of the composite gels.

### 2.2. The Oscillatory Frequency Sweep

Dynamic rheological properties are a critical indicator for characterizing the internal structural evolution of gels, where G′ and G″ correspond to the elastic and viscous behaviors of the material, respectively [17]. Experimental data showed that during frequency sweep tests, both G′ and G″ exhibited a frequency-dependent increase, with G′ consistently higher than G″ (Figure 1B). This indicated that the composite hydrogel possesses distinct solid-like characteristics. The sharp upward trend of both moduli at higher frequencies may be attributed to the enhanced intermolecular interactions between Sanxan and YP. Furthermore, the significant influence of frequency on the modulus values suggested that the continuous phase network of the gel system is primarily maintained by non-covalent interactions, such as hydrogen bonding and hydrophobic interactions [18].

Tan δ (defined as G″/G′) serves as an essential indicator for differentiating fluid and gel states: values exceeding 1 signify a dilute solution, while the range 0 < tan δ < 1 denotes a weak gel system [19]. In this study, tan δ values of the gel remained consistently within the 0.1–0.3 range (Figure S3), consistent with weak gel rheological properties. Notably, data indicate that food gels exhibiting tan δ values between 0.1 and 1 offer enhanced safety for dysphagia patients during ingestion. However, holistic assessment incorporating supplementary parameters such as gel texture and yield stress remains necessary.

Results from model fitting indicated the following (Table 2): In the low-frequency region (0.1–1 rad/s), the elastic modulus coefficient (a′) ranged from 132.7 to 248.9. The SYPH sample exhibited the highest elastic strength (a′ = 248.9), while SYPC showed the lowest (a′ = 132.7). The corresponding frequency exponent (b′) was very low (0.028–0.054), approaching the zero-frequency dependence characteristic of an ideal elastic solid. As the frequency increased to the range of 1–100 rad/s, the rheological properties evolved significantly: the elastic frequency exponent b′ rose to 0.037–0.116, and the viscous frequency exponent b″ further increased to 0.134–0.198, indicating that the viscous response was more sensitive to frequency changes at high frequencies. Notably, SYPD and SYPH retained the highest modulus values across both low and high frequencies (a′_SYPD_ = 229.2; a′_SYPH_ = 248.9), which may suggest a more stable three-dimensional network or enhanced intermolecular associations in these formulations. Materials with low b′ values (b′_SYPG_ = 0.028) demonstrated better stability during low-frequency processing, whereas high a″ values (a″_SYPH_ = 22.99) facilitated smooth extrusion. The fitted parameters of the viscous modulus are relatively low, a common observation in rheological studies where most literature prioritizes the elastic modulus to characterize network stiffness; however, the retained viscous modulus values remain reliable for comparing dissipation behavior across SPYs.

### 2.3. The Dynamic Temperature Sweep

**Figure 2:**
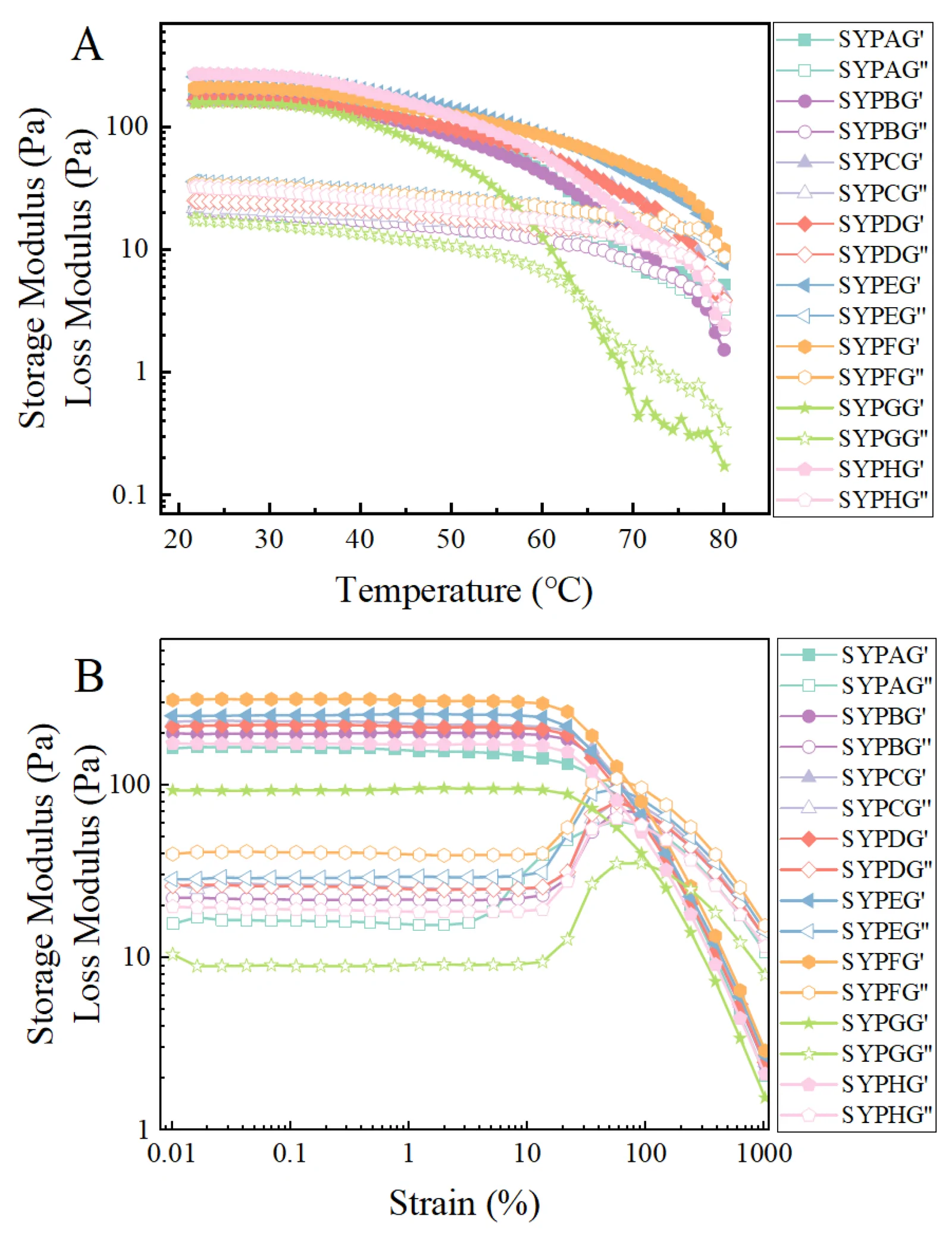
(**A**) Temperature-dependent curves of SYPs hydrogels during 20–80 °C; (**B**) amplitude sweep curves at 0.01–1000% strain.

The effect of temperature variation on the rheological properties of the gel was systematically investigated via a temperature sweep test ranging from 20 to 80 °C. The experimental data showed that the elastic modulus remained stable within the 20–40 °C range (Figure 2A), indicating good thermal stability of the gel network in this temperature domain. As the temperature exceeded 40 °C, G′ exhibited a significant downward trend. Notably, an intersection point between G′ and G″ was observed in the 65–80 °C region, a rheological characteristic that clearly signifies the gel–sol phase transition. Importantly, the YP-modified composite gels displayed a higher phase transition temperature, a phenomenon consistent across both high- and low-concentration groups. This observation suggests that the incorporation of YP may contribute to improving the thermal stability within the present system. However, given the indirect nature of the rheological evidence, further studies using complementary techniques (e.g., DSC, TGA, or structural analysis) would be beneficial to confirm this effect and support material development.

The temperature sweep data were fitted using the Arrhenius equation to derive three key parameters: lnA (natural logarithm of the Arrhenius constant), Eα (activation energy for gel flow), and R^2^ (coefficient of determination) [20]. The results showed that all gel samples exhibited negative lnA values, indicating a low baseline viscosity at high temperatures (Table 3). Specifically, the pure Sanxan sample (SYPG) showed the lowest lnA value (−25.18), whereas the composite system with 20% YP and 2% Sanxan (SYPF) displayed the highest lnA value (−2.15). This phenomenon suggested that at theoretically infinite temperatures, intermolecular interactions were weak, resulting in excellent high-temperature fluidity. The activation energy (Eα) ranged from 31,799.4 to 89,561.7 J·mol^−1^. The pure Sanxan sample (SYPG) had the highest Eα (89,561.7 J·mol^−1^), while the SYPF composite had the lowest (31,799.4 J·mol^−1^), indicating that the yeast protein-containing composite gel requires lower energy for flow, which helps reduce energy consumption during processing. Overall, as yeast protein content increased from 5% to 20%, the absolute value of lnA exhibited a significant decrease, while Eα progressively declined. Similarly, increasing the Sanxan content from 1.5% to 2% resulted in the same parameter variation pattern. Notably, the pure Sanxan sample exhibited relatively high Eα and low lnA values, which may be tentatively attributed to the absence of protein components—although this interpretation remains speculative and requires further investigation.

### 2.4. The Strain Sweep

Strain amplitude sweep tests identified the linear viscoelastic region (LVR), enabling systematic analysis of the gels’ viscoelastic properties and structural stability. Figure 2B demonstrates significantly higher G′ than G″ at low strain, confirming elastic-dominated behavior. Increasing YP content induced a gradual enhancement in G′ values—consistent with frequency sweep observations. Under identical strain conditions, both moduli tended to increase with YP concentration, suggesting that YP may contribute to improving the mechanical strength of the gel network. These findings are corroborated by reports on oleogel systems [21].

All gel samples exhibited a plateau region for G′ and G″ within the LVR. Beyond the linear viscoelastic region, G′ progressively decreased with increasing strain amplitude, signifying inter-cycle strain softening and progressive gel network disruption. Conversely, G″ initially rose to a discernible peak before declining at higher strains. This combination of diminishing G′ and G″ overshoot aligns with Type III nonlinear viscoelastic behavior as classified by Hyun et al. [22], commonly designated as weak strain overshoot. At still higher strains, G″ exceeded G′, indicating a transition from an elasticity-dominated gel to a viscosity-dominated flowing state. During this phase transition, G″ displayed a “Type III nonlinear behavior” characterized by a peak followed by a decline [23], indicating strain softening and progressive yielding of the gel network. This dynamic response enhances the material’s applicability in 3D printing contexts: it temporarily softens during extrusion to facilitate shaping and rapidly recovers rigidity to maintain structural integrity [24]. The experiments revealed a critical point where G′ equals G″ for all gels; notably, higher YP concentrations corresponded to larger critical strain values at this crossover point, suggesting that high YP content improves structural stability under large deformations. Furthermore, all systems exhibited typical shear-thinning behavior beyond the LVR, with modulus reduction patterns consistent with non-Newtonian fluid characteristics. This series of rheological characterizations provides a crucial theoretical basis for regulating the mechanical properties and processability of the gels.

### 2.5. LAOS

**Figure 3:**
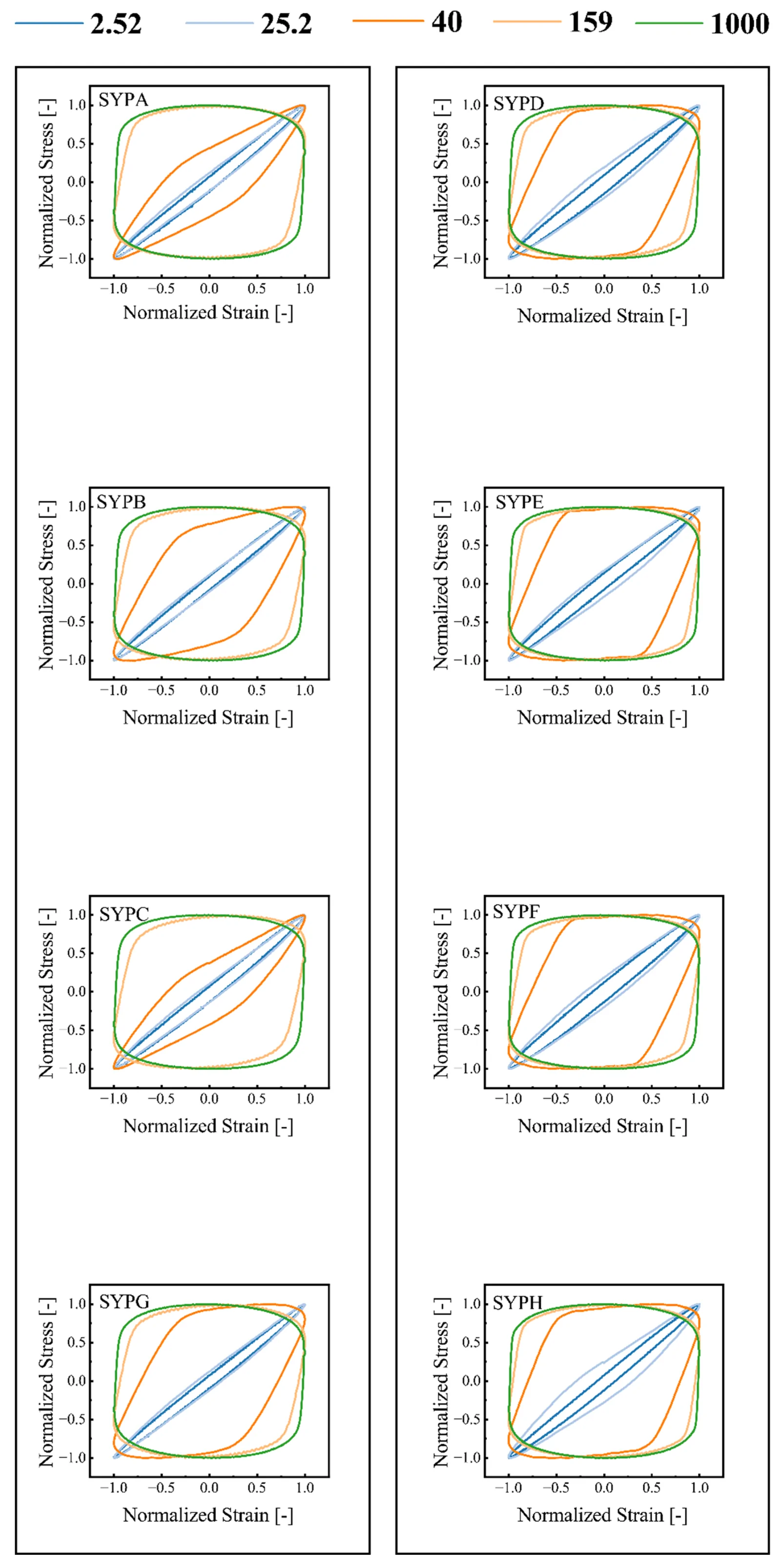
Elastic Lissajous curves of SYPs hydrogel at various strain amplitudes (2.52%, 25.2%, 40%, 159%, and 1000%).

Lissajous curves effectively characterize the structural transition properties under large deformations by depicting the functional relationship between intracyclic stress and applied strain/shear rate. This technique not only provides an intuitive visualization of nonlinear behavior but also reveals critical structural information [25,26]. Initial G′ and G″ exhibited stability across strain amplitudes, indicating dynamically balanced molecular interactions (resisting shear deformation) and corresponding Lissajous curves displaying stable closed elliptical forms [27]. Upon entering the nonlinear region, the material moduli showed significant strain dependence, leading to characteristic changes in the curve morphology (Figure 3). Notably, the specific morphology of the Lissajous curve was strictly dependent on the material type and the applied stress level. In the LVR, the curve appeared as a typical elastic-dominated ellipse, which was consistent with the linear viscoelastic solid behavior and high stiffness observed in SAOS tests (Figure 2B). As strain increased, the curve gradually distorted into a quasi-parallelogram configuration, a morphological evolution directly correlated with the material’s microstructural characteristics [28]. The transition from a broad ellipse to a parallelogram essentially reflected the enhancement of viscous dissipation mechanisms during cyclic deformation and a shift from elastic to viscous dominance. Specifically, the continuous expansion of the ellipse area with increasing strain suggested intensified energy dissipation and an increased tendency for gel structure disruption [29]. Compared to the low-concentration groups, the composite hydrogels containing 5% and 20% YP (particularly the 5% sample) exhibited minimal variation in ellipse area within the strain range of 2.52–40%. In contrast, the gel without YP showed a more pronounced deformation response. This directly confirmed that the unmodified gel possesses poorer microstructural stability and was more susceptible to structural breakdown under large deformations, resulting in a stronger nonlinear response.

**Figure 4:**
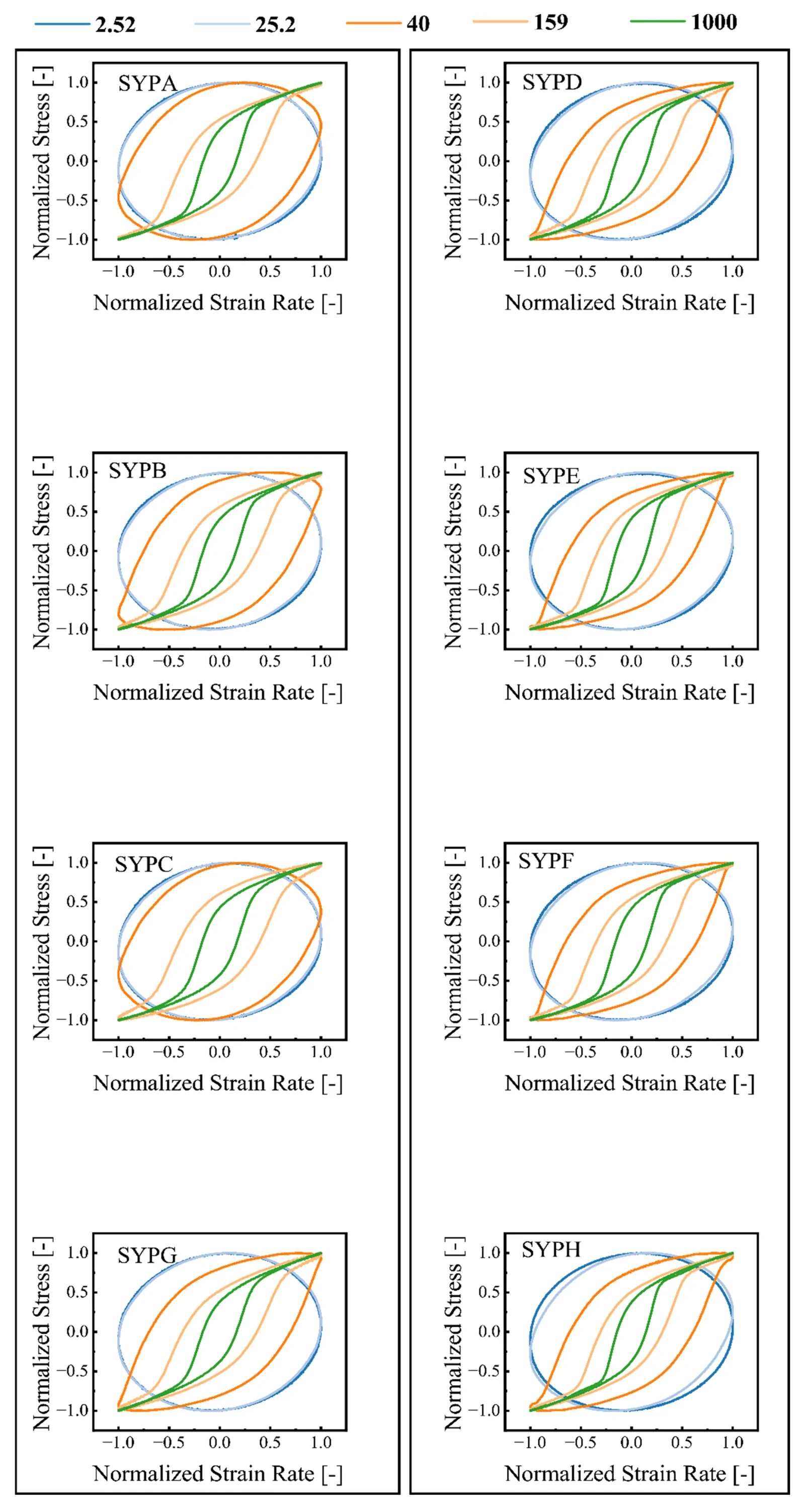
Viscous Lissajous curves of SYPs hydrogel at various strain amplitudes (2.52%, 25.2%, 40%, 159%, and 1000%).

With increasing strain amplitude, the curve shape gradually evolved from an initial ellipse to a spindle-like configuration, accompanied by a decreasing trend in the enclosed loop area. This morphological change visually reflected the shear-thinning characteristic of the gel system, which is of great value for the extrusion process of 3D printing inks, ensuring smooth extrusion [30]. When the shear stress and strain rate loops transformed from an elliptical shape to an approximate parallelogram, it indicated significant nonlinear viscoelastic behavior in the system (Figure 4). Notably, the gel exhibited a strong shear-thinning effect at higher strain rates. For low-concentration Sanxan composite gels, samples with 5% and 20% YP showed only slight deformation and insignificant changes in loop area within the strain range of 2.52% to 40%. In contrast, the 10% YP sample and the gel without YP exhibited distinct nonlinear viscous component characteristics near 25.2% strain, a phenomenon clearly identifiable through changes in curve slope and morphological distortion. The elastic Lissajous curve features of the 5% and 20% YP gels suggested that these systems tend to exhibit a solid-like mechanical response dominated by elasticity. This finding provided an important theoretical basis for regulating the rheological properties of the gels.

**Figure 5:**
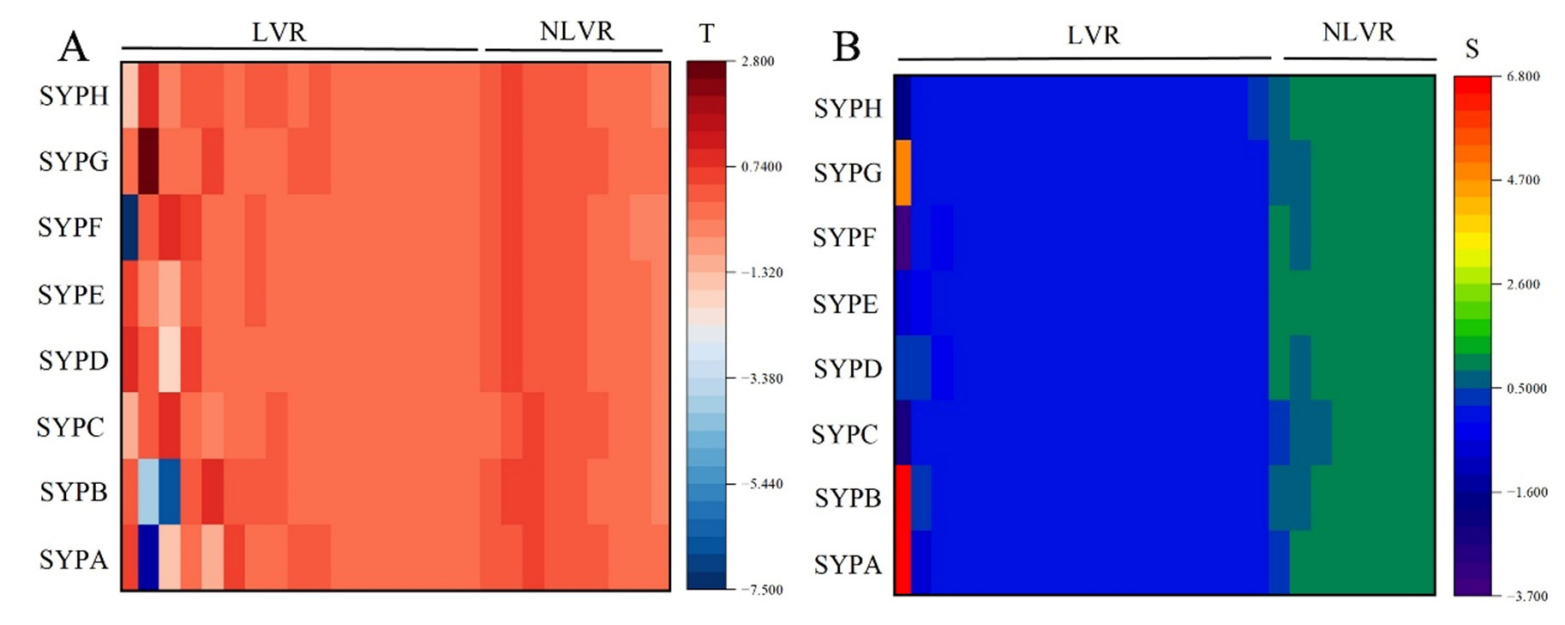
(**A**) Shear thickening ratio (T factor) and (**B**) the strain stiffening ratio (S factor) of different composite hydrogels.

Shear-thickening ratio (T factor) and the strain-stiffening ratio (S factor) are two key parameters for quantifying the rheological behavior of gels (Figure 5). The T-factor characterizes the degree to which viscosity increases with shear rate, reflecting the shear-thickening nature of the material; the S-factor describes the gel’s resistance to strain, embodying its strain-hardening behavior. Analysis of experimental data revealed that the T-factor exhibited a non-monotonic trend of increasing first and then decreasing with strain: the initial rise may be attributed to the disruption of protein–polysaccharide chain entanglements or an increase in cross-linking points, leading to a denser structure at higher shear rates; the subsequent decline indicated weakened intermolecular interactions and enhanced system fluidity [31]. The variation pattern of the S-factor shows that its value approaches zero in the low-strain region, confirming that the gel primarily exhibits linear viscoelasticity. As strain increased, the absolute value of the S-factor rose significantly, indicating that the material had entered a nonlinear deformation stage [32]. This strain-hardening phenomenon was directly correlated with the reinforcement of the internal cross-linked network structure, suggesting the formation of a more robust 3D network architecture during deformation [33].

## 3. Conclusions

This study developed a composite hydrogel system incorporating YP and the microbial anionic polysaccharide sanxan. Systematic characterization revealed enhanced thermal stability through 5–15 °C heat resistance improvement with YP addition. Rheologically, all samples exhibited frequency-dependent weak-gel behavior with elastic dominance under small-amplitude shear. Elevated storage moduli in YP-containing gels indicated enhanced network rigidity. Amplitude sweeps demonstrated YP concentration-dependent shifts in the G′ = G″ crossover toward higher strains, suggesting improved resistance to network disruption. Lissajous analysis showed: standard elliptical curves at low strain (linear viscoelastic behavior); parallelogram transformation at high strain (increased viscous dissipation); and strain-induced curve area expansion indicating intensified energy dissipation. In low-concentration systems, SYPA/SYPC gels maintained stable elliptical areas under high strain, outperforming pure sanxan. YP appears to contribute to increased network rigidity, as suggested by the S-factor/T-factor analysis.

A primary advantage of this work is the integrated application of TGA, DSC, SAOS, amplitude sweeps, LAOS, Lissajous analysis, and S-T factor mapping, enabling multi-scale gel assessment across temperature, frequency, and strain domains. Although this study primarily elucidates the influence of compositional ratios on the rheological behavior of Sanxan–yeast protein hydrogels, the molecular interactions, particularly hydrogen bonding, hydrophobic association, and electrostatic attraction, underlying the formation and stability of the three-dimensional network remain a fundamental determinant of macroscopic functionality. Investigating these interactions represents not only a critical scientific frontier but also a central focus of our forthcoming research endeavors. Two limitations require attention: direct spectroscopic/microscopic evidence for protein–polysaccharide interactions is absent; tested YP and sanxan concentration ranges were limited. Future work should combine FTIR (or comparable spectroscopy) with microscopic characterization, structural recovery assays, long-term stability monitoring, and application-specific performance verification to confirm the proposed mechanism and determine optimal practical formulations.

## 4. Materials and Methods

### 4.1. Materials

Sanxan was sourced from Hebei Xinhe Biochemical Co., Ltd. (Model: SZ-400, Xingtai, China), produced via fermentation with *Sphingomonas sanxanigenens* NX02. The polysaccharide exhibits a weight-average molar mass (Mw) of 408 kDa and number-average molar mass (Mn) of 382 kDa, yielding a polydispersity index (PDI) of approximately 1.07, indicative of a narrow molecular weight distribution. This characteristic may be associated with the preparation process and purification. The YP (Model: Anpro 2020) used in this study was supplied by Angel Yeast Co., Ltd. (Yichang, China) and had a protein content of 81 wt%, as determined in accordance with GB 5009.5-2025 [34].

### 4.2. Preparation of SYP Hydrogels

Firstly, YP and sanxan powders were individually dissolved in deionized water to prepare stock solutions at varying concentrations, as specified in Table 4 (YP: 5%, 10%, and 20%, *w*/*v*; sanxan: 1.5% and 2%, *w*/*v*). Subsequently, these stock solutions were mixed at an optimal volume ratio of 1:5 (*v*/*v*, YP stock solution: sanxan stock solution), which was determined by preliminary experiments. The mixture was then mechanically stirred for 5 min to obtain a homogeneous composite solution (Table 4). The YP–sanxan mixture was heated at 75 °C for 30 min as a controlled thermal-processing step and subsequently cooled to room temperature to promote gel formation. Because this treatment may induce partial unfolding or aggregation of YP, the resulting system is considered a heat-treated YP–sanxan composite gel rather than a gel containing fully native YP. The resulting hydrogel samples were designated as the SYPA-SYPH series. Specifically, samples SYPA-SYPC constituted the low-concentration sanxan composite group, whereas samples SYPD-SYPF constituted the high-concentration sanxan composite group. Samples SYPG and SYPH served as blank controls. Gel formation was verified using the standard test tube inversion method, where the absence of significant flow within 1 min of inversion was defined as the criterion for successful gelation.

### 4.3. Thermodynamic Characterization

#### 4.3.1. Thermogravimetric Analyses (TGA)

TGA was employed to evaluate the thermal degradation profile of freeze-dried hydrogels. Samples (1.0 mg ± 0.5 mg) were precisely weighed and placed in aluminum crucibles (100 μL). The experiment utilized a nitrogen flow of 20 mL/min and a heating program linearly increasing temperature from 20 °C to 300 °C at 20 °C/min.

#### 4.3.2. Differential Scanning Calorimetry (DSC)

DSC measurements were conducted on freshly made hydrogels (100 µL sample volume) loaded into perforated aluminum crucibles. The micro-apertures in the lids facilitated gas exchange for equilibrium. The temperature protocol cycled linearly: cooling from room temperature to −30 °C and subsequent heating to 40 °C, each segment at a constant 10 °C/min rate. Oxidative interference was minimized by maintaining a 20 mL/min nitrogen flow.

### 4.4. Rheological Measurements

An Anton Paar MCR 302 rheometer(Anton Paar, GmbH, Ostfildern, Germany), fitted with parallel plate (50 mm, 1 mm gap) and cone-plate (50 mm, 1° cone angle, 0.1 mm gap) geometries, characterized the rheology of SYPs composite hydrogels. Testing occurred at 25 °C (excluding temperature sweeps), following a 2-min sample equilibration period to achieve a stable state.

#### 4.4.1. The Oscillatory Frequency Sweep

Frequency sweep tests with a fixed strain amplitude of 0.1% were performed across a 0.1–100 rad/s frequency range to characterize the gel properties under varying physical conditions. Additionally, the power-law (PL) equation was employed to establish the correlation between angular frequency (ω) and the storage (G′) and loss (G″) modulus.

(1) $$G^{\prime }=a\prime \omega^{b^{\prime }}$$

(2) $$G^{\prime \prime }=a\prime \prime \omega^{b^{\prime \prime }}$$

#### 4.4.2. The Dynamic Temperature Sweep

To investigate the temperature-dependent properties of the gels, temperature sweep tests were conducted by heating the samples from 20 to 80 °C. Notably, to prevent water evaporation, the edges of the parallel plate geometry were sealed with silicone oil. The resulting data were then fitted to the Arrhenius equation:

(3) $$\eta =Aexp\left(E\alpha ∕RT\right)$$

#### 4.4.3. The Strain Sweep

Strain sweep tests at a fixed angular frequency of 10 rad s^−1^ and 25 °C employed strain amplitudes spanning 0.01% to 1000%, thereby identifying the linear viscoelastic region (LVR) and flow point of the composite gels—a critical step for directing subsequent research.

#### 4.4.4. LAOS

LAOS tests were conducted as strain sweeps at a fixed angular frequency of 1 rad/s over a strain range of 0.01% to 1000%. Raw waveform data were collected in transient mode and subsequently processed using Origin 2024 software for normalization and area calculation. Based on Equations (4) and (5), the strain-stiffening ratio (S-factor) and shear thickening ratio (T-factor) were calculated.

(4) $$S=\frac{{(G}^{\prime }L-G\prime M)}{G\prime L}$$

(5) $$T=\frac{\eta \prime L-\eta \prime M}{\eta \prime L}$$

In Equation (4), G’L represents the storage modulus at large strain, and G’M denotes the minimum storage modulus. In Equation (5), η’L is the complex viscosity at a high shear rate, while η’M is the complex viscosity at the minimum shear rate [35].

## Figures and Tables

**Table 1:** Data obtained from TGA and DSC curves.

Sample	TGA		DSC	
	Weight Loss (%)	T Onset (°C)	T Freezing (°C)	Enthalpy (J/g)
SYPA	90.1	223.2	−18.5	288.1
SYPB	89.4	227.6	−18.2	250.9
SYPC	92.3	230.4	−17.4	266.5
SYPD	86.2	220.5	−16.8	297.4
SYPE	87.1	227.1	−18.9	271.9
SYPF	89.9	229.6	−16.5	277.2
SYPG	86.9	217.5	−19.3	249.7
SYPH	82.4	214.0	−17.6	275.8

**Table 2:** Fitting parameters of the PL model derived from frequency sweep data.

Samples	G′ = a′ω^b′^			G″ = a″ω^b″^		
	a′	b′	R^2^	a″	b″	R^2^
Low frequency (0.1–1 rad s^−1^)						
SYPA	137.9	0.034	0.996	10.81	0.095	0.811
SYPB	132.9	0.037	0.997	11.39	0.078	0.899
SYPC	132.7	0.039	0.991	12.55	0.088	0.818
SYPD	229.2	0.048	0.997	22.67	0.078	0.875
SYPE	144.6	0.049	0.988	14.26	0.097	0.888
SYPF	152.8	0.054	0.998	16.79	0.087	0.759
SYPG	135.1	0.028	0.995	11.00	0.108	0.991
SYPH	248.9	0.032	0.998	22.99	0.088	0.974
High frequency (1–100 rad s^−1^)						
SYPA	137.5	0.053	0.996	9.95	0.169	0.947
SYPB	129.5	0.072	0.989	10.78	0.161	0.957
SYPC	119.5	0.116	0.841	11.69	0.161	0.951
SYPD	215.5	0.096	0.933	22.44	0.134	0.954
SYPE	141.4	0.074	0.991	13.14	0.162	0.908
SYPF	157.4	0.049	0.827	16.48	0.139	0.941
SYPG	137.1	0.037	0.901	9.62	0.198	0.901
SYPH	251.2	0.042	0.907	22.02	0.143	0.971

**Table 3:** Fitting parameters for the Arrhenius equation were extracted from dynamic temperature sweep data.

Sample Label	lnA	Ea (J mol^−1^)	R^2^
SYPA	−10.58	52,708.3	0.847
SYPB	−10.38	52,221.9	0.850
SYPC	−5.19	38,930.3	0.805
SYPD	−5.49	39,792.5	0.817
SYPE	−4.38	37,960.9	0.868
SYPF	−2.15	31,799.4	0.854
SYPG	−25.18	89,561.7	0.859
SYPH	−10.57	53,772.5	0.871

**Table 4:** SYPs Hydrogel Preparation Component Table.

Sample	Yeast Protein (%, *w*/*v*)	Sanxan (%, *w*/*v*)	Ratio (*v*:*v*)
SYPA	5	1.5	1:5
SYPB	10	1.5	1:5
SYPC	20	1.5	1:5
SYPD	5	2	1:5
SYPE	10	2	1:5
SYPF	20	2	1:5
SYPG	-	1.5	1:5
SYPH	-	2	1:5

## Data Availability

All data presented in this work are available from the corresponding author upon reasonable request. For further information or specific data inquiries, please contact the corresponding author directly.

## Supplementary Materials

The following supporting information can be downloaded at: https://www.mdpi.com/article/10.3390/gels12080747/s1. Figure S1: TGA of Yeast Protein; Figure S2: DSC of hydrogels; Figure S3: the tan δ values of the gels (tan δ = G″/G′); Figure S4: pH and conductivity charts for precursor solution hydrogels and Yeast Protein solution.
