# Supplementary material for: Rheological Characterization of Yeast Protein–Sanxan Composite Hydrogels via SAOS, LAOS and Thermal Analysis
Source: Gels. 2026 Aug 20;12(8):747. doi: 10.3390/gels12080747 (PMC13512834; doi:10.3390/gels12080747)
Supplement: Supplementary file 1 [file gels-12-00747-s001.zip › gels-4452250-supplementary.pdf]

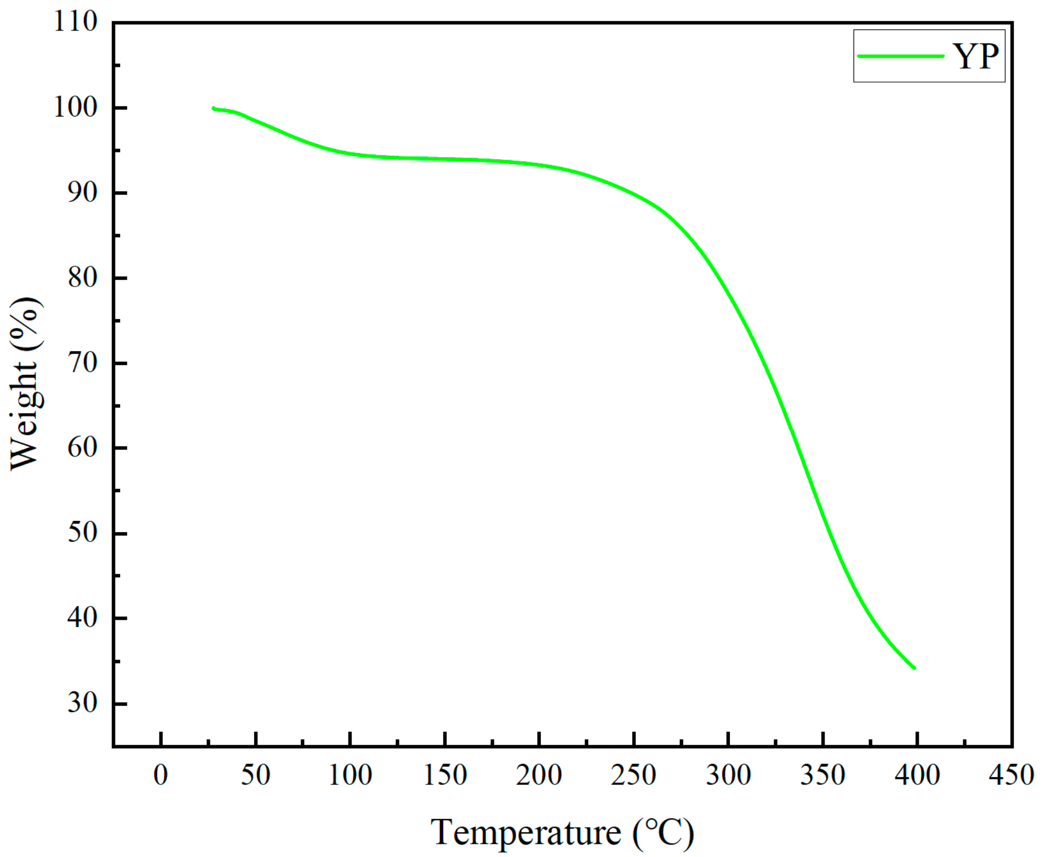

Figure S1. TGA of Yeast Protein.

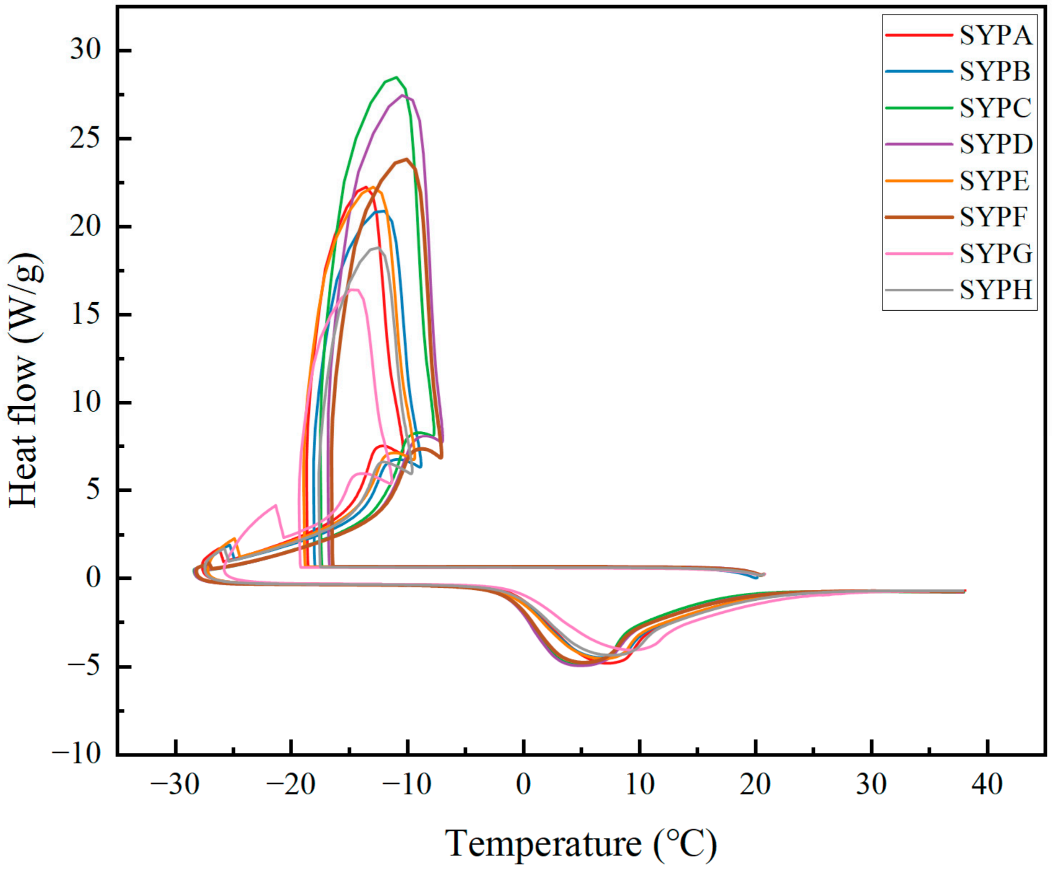

Figure S2. DSC of hydrogels.

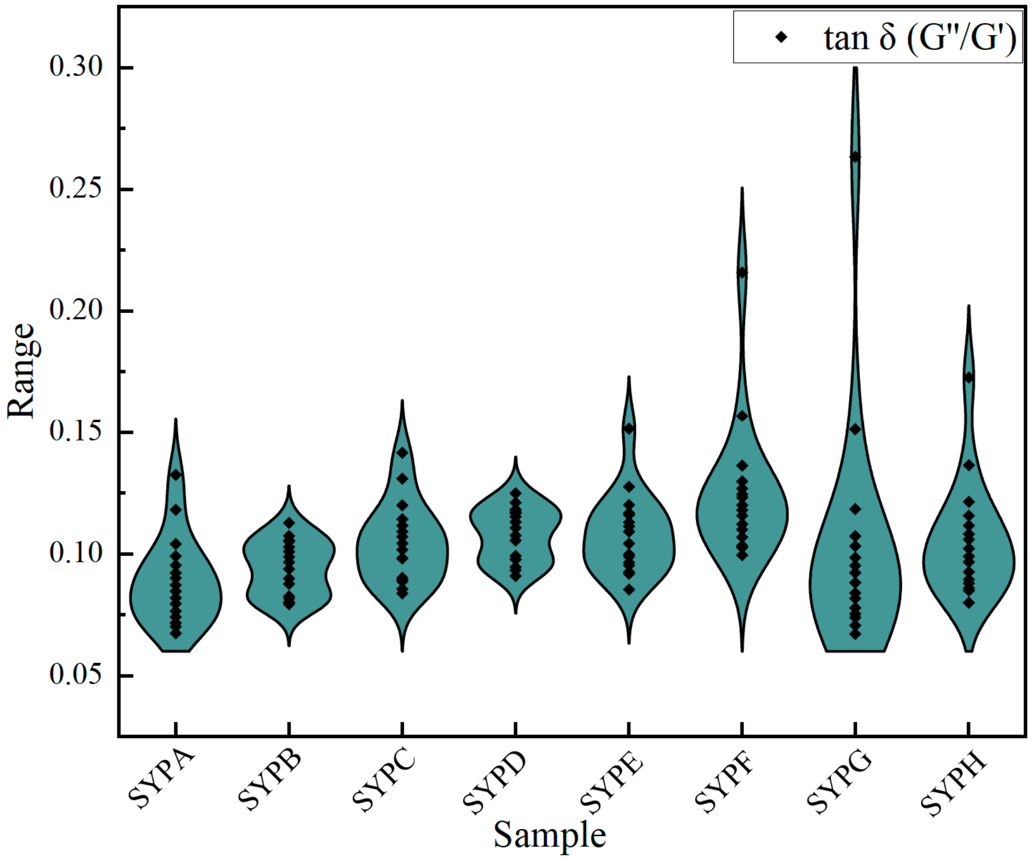

Figure S3. The tan δ values of the gels (tan δ = G''/G').

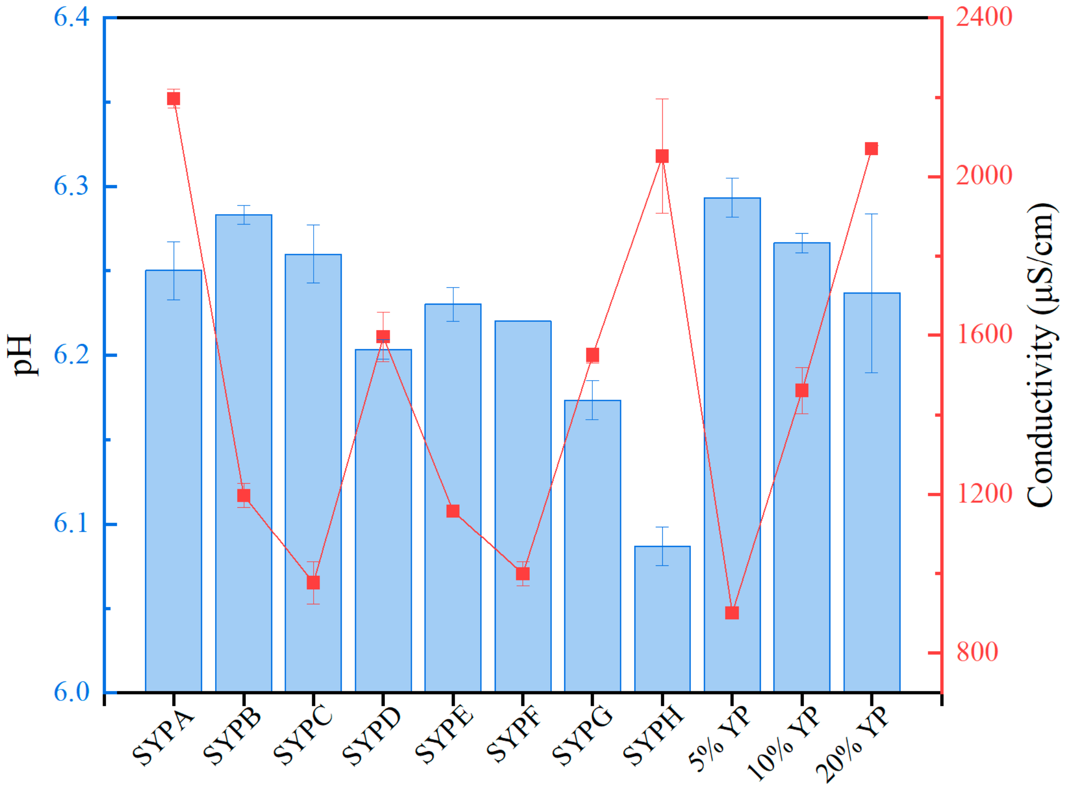

Figure S4. pH and conductivity charts for precursor solution hydrogels and Yeast Protein solution.

Figure S4 displays the pH and conductivity values of various gel precursor solutions and YP solutions at different concentrations. The data show that the pH values remained relatively stable for all precursor solutions before and after the addition of YP, indicating that mixing YP with sanxan gum does not significantly alter the system pH. Regarding conductivity, the values for YP solutions alone increased with increasing concentration. However, after mixing with sanxan gum, the gel precursor solutions exhibited a different trend: the conductivity gradually decreased with increasing YP concentration for both the low- and high-concentration precursor groups. This phenomenon may be attributed to ionic interactions: the YP solution itself may interact with the negatively charged sanxan gum, leading to a reduction in either the number of free ions or their mobility within the system, thereby causing the conductivity to decrease. Furthermore, for precursor solutions with different sanxan gum concentrations, significant differences were evident among the low-concentration groups, whereas the differences were relatively smaller among the high-concentration

groups. This concentration-dependent pattern of conductivity variation is closely linked to the degree of polymer network entanglement and its stabilizing effect on encapsulated substances.
